# PLL Based Energy Efficient PV System with Fuzzy Logic Based Power Tracker for Smart Grid Applications

**DOI:** 10.1155/2016/2708075

**Published:** 2016-05-15

**Authors:** G. Rohini, V. Jamuna

**Affiliations:** Department of Electrical and Electronics Engineering, Jerusalem College of Engineering, Anna University, Chennai 600100, India

## Abstract

This work aims at improving the dynamic performance of the available photovoltaic (PV) system and maximizing the power obtained from it by the use of cascaded converters with intelligent control techniques. Fuzzy logic based maximum power point technique is embedded on the first conversion stage to obtain the maximum power from the available PV array. The cascading of second converter is needed to maintain the terminal voltage at grid potential. The soft-switching region of three-stage converter is increased with the proposed phase-locked loop based control strategy. The proposed strategy leads to reduction in the ripple content, rating of components, and switching losses. The PV array is mathematically modeled and the system is simulated and the results are analyzed. The performance of the system is compared with the existing maximum power point tracking algorithms. The authors have endeavored to accomplish maximum power and improved reliability for the same insolation of the PV system. Hardware results of the system are also discussed to prove the validity of the simulation results.

## 1. Introduction

The steady increase in demand for power supply with overexploited decreasing conventional sources increases the necessity for alternative energy sources. Photovoltaic (PV) array, using solar energy as a source, is a better option as it provides a pollution-free, easily available, clean energy source [1]. For a smart grid application, PV array can be used as a source of energy in conjunction with the existing conventional source and wind energy. A photovoltaic system converts photon energy from sunlight into electricity. With PV panel prices dropping and the desire to “go green” expanding, demand for PV power is improving. As the efficiency of a PV array is much less, it becomes imperative to extract the maximum available power out of it. Various maximum power point tracking (MPPT) techniques are available to extract maximum power available from the solar energy [2–6]. According to maximum power transfer theorem, if the source impedance is equal to the load impedance, maximum energy can be transferred from the source to the load. The inclusion of DC-to-DC converter with MPPT technique varies the equivalent impedance, thereby matching the load resistance to achieve maximum power transfer. The perturb and observe (P&O) method is the simplest method. Maximum power control is achieved by forcing the derivative of the power to be equal to zero under power feedback control [7, 8]. But this method produces oscillations closer to the maximum power point, thereby raising the response time. Incremental conductance (INC) process traces the maximum power by observing the conductance. It uses *dI*/*dV* and compares it with *I*/*V*, thereby adjusting the step size to track the maximum power point of PV array [9, 10]. Incremental resistance (INR) is similar to INC except that it compares *dV*/*dI* with *V*/*I* for tracking maximum power point [11]. Fuzzy logic control is an artificial intelligence technique which can be used to track the maximum power point to improve the performance of the system. As there is fuzziness in the input for PV array like change in insolation, temperature, and shading effect during the entire day, this logic is more suitable than the other methods for tracking maximum power point. In addition, this system does not require the knowledge of the exact model [12–17]. To transmit the power at the required voltage and current level from the PV array to the load, boost converter is used [18]. The interleaved soft-switching boost converter (ISSBC) shares the input current all along each phase, thereby decreasing the current rating of the switching device. The ripple content present in the input current and the output voltage and the size of the passive components are also reduced in this converter [19, 20]. The problems caused due to EMI, switching loss, and diode recovery loss can be overcome by using the resonant soft-switching technique [21–24]. This technique does have certain limitations. The fidelity of the PWM signal to the analog input is determined by the linearity of the ramp-wave signal. The performance of the comparator determines speed, accuracy, and jitter. Phase-locked loops (PLLs) synchronize a local voltage-controlled oscillator (VCO) to an external frequency input by means of an electronic servo loop [25]. It is used with converter to achieve constant operating frequency over a wide output voltage range, eliminating the dependence of switching frequency on duty cycle or voltage conversion range [26].

In the present work, to improve the dynamic performance of the PV system, cascaded converter topology is used with intelligent power tracker and modified control strategy. The first conversion stage uses the fuzzy logic controller to track the maximum power. The simulation is performed for three algorithms in comparison with fuzzy based MPPT whereas in the literature comparison of fuzzy logic with any one algorithm is available. The phase-locked loop is used in the cascaded three-stage converter to extend the soft-switching region to minimize the losses. The interleaved soft-switching technique available in the literature is extended in the present work for three-stage converter for improved efficiency. In addition, the power handling capability is improved and the ripple content at the output is reduced.

The PLL control scheme prevents the operating frequency of the circuit from being lower than the resonant frequency.* In this work*, phase-locked loop is implemented to maintain the required frequency of operation with reduced ripple and to increase the soft-switching range of operation. The PV array was mathematically modeled using the basic equations to match the actual parameters of the nonlinear model. The performance of the system is compared with the other conventional algorithms. The simulation is performed using the Simulink software and the hardware results are found to be in line with the simulation results.

This paper is organized as follows.  Section 2 presents system description. Mathematical model of solar PV module is discussed in Section 3. In Section 4, fuzzy logic based maximum power tracker used in the present work is discussed.  Section 5 presents design of three-stage soft-switching boost converter.  Section 6 describes the phase-locked loop for extending the soft-switching region.  Section 7 deals with results and discussions. Finally, Section 8 summarizes the work done as conclusions.

## 2. System Description

**Figure 1:**
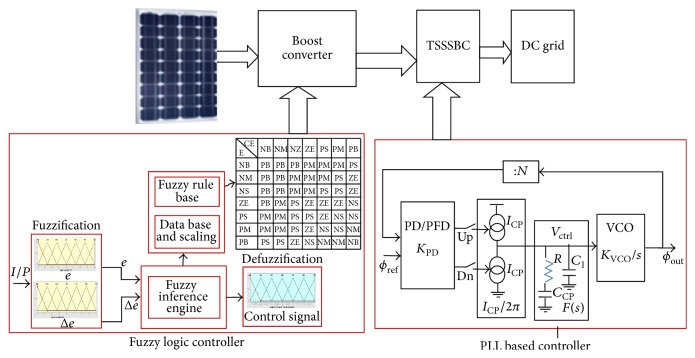
Block diagram of cascaded converter fed PV array connected to grid.

Block diagrammatic representation of the PLL based energy efficient PV system with intelligent power tracker and cascaded converter is shown in Figure 1. The system consists of PV array acting as the source which converts light energy into electrical energy. In this work, both feedforward and feedback control are performed. Maximum power point tracking is performed as feedforward control by using fuzzy logic controller for the first converter in the cascaded network. Driving pulses to the switches are generated using the fuzzy based power tracking algorithm. Three-stage soft-switching boost converter (TSSSBC) is used for the second stage of cascaded converter to match the load requirements. Pulses for the TSSSBC are 120° phase shifted from each other. A three-stage boost converter is designed, developed, and modified from a normal boost converter. As the number of stages in the boost converter is increased, the size of the inductor is reduced, so that it could be made compact and reduction in ripple content could also be achieved. The phase delay for each stage is given by 360/*N*, where *N* is the number of stages. As the number of stages increases, the quality of regulated output increases, but, at the same time, the complexity of the circuit also increases. To ensure reduced complexity with reduced ripple content, a three-stage boost converter is used in the present work. To ensure accurate terminal voltage with lower ripple content, feedback control with phase-locked loop is proposed in the present work.

## 3. Mathematical Model of Solar PV Module

**Figure 2:**
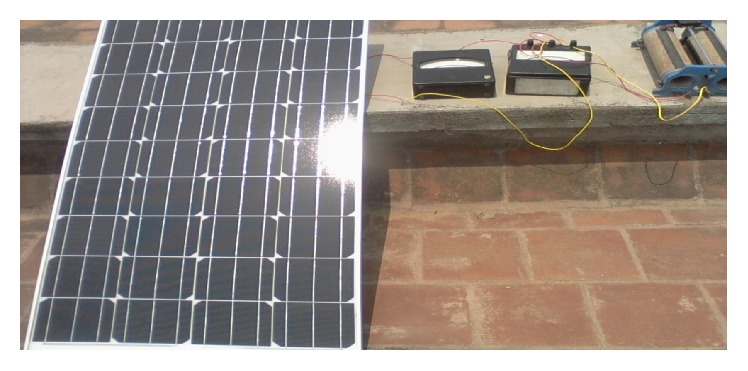
Photovoltaic panel under testing.

Use of equivalent circuits makes it possible to model the characteristics of a PV cell [27–29]. A single diode model for PV array is considered in this work. The model for PV panel is developed as per the specifications given in Table 1 and the same is considered for simulation using the MATLAB/Simulink package. The test setup of the PV panel is shown in Figure 2.

The basic equation that mathematically describes the *I*-*V* characteristics of an ideal PV cell is

(1) $$\begin{array}{c} I=I_{PV,cell}-I_{D}, \end{array}$$

where *I*_PV,cell_ is the current generated by the PV panel and *I*_*D*_ is the diode current. Substituting for *I*_*D*_, we get

(2) $$\begin{array}{c} I=I_{PV}-I_{0}\left[\;\exp\left(\;\frac{qV}{akT}\;\right)-1\;\right], \end{array}$$

where *I*_0_ is the reverse saturation current.

The basic equation does not represent the actual characteristic of the array and hence requires inclusion of additional parameters of equivalent series (*R*_*s*_) and parallel resistance (*R*_*p*_). Hence,

(3) $$\begin{array}{c} I=I_{PV}-I_{0}\left[\;\exp\left(\;\frac{V+IR_{s}}{a\cdot V_{t}}\;\right)-1\;\right]-\left(\;\frac{V+I\cdot R_{s}}{R_{p}}\;\right), \end{array}$$

where *V*_*t*_ is the thermal voltage.

The photon generated current is also influenced by the temperature according to the following equation:

(4) $$\begin{array}{c} I_{PV}=\left(\;I_{PV,n}+K_{I}\Delta_{T}\;\right)\frac{G}{G_{n}}, \end{array}$$

where *I*_PV,*n*_ is the light generated current at nominal condition; Δ*T* = *T* − *T*_*n*_, where *T* and *T*_*n*_ are actual and nominal temperatures in kelvin; *G* is the irradiation on the panel; and *G*_*n*_ is the nominal irradiation. The diode saturation current *I*_0_ is given by

(5) $$\begin{array}{c} I_{0}=I_{0,n}{\left(\;\frac{T_{n}}{T}\;\right)}^{3}\exp\left[\;\frac{qE_{g}}{ak}\left(\;\frac{1}{T_{n}}-\frac{1}{T}\;\right)\;\right], \end{array}$$

where *E*_*g*_ is the energy gap and *I*_0,*n*_ is the nominal saturation current:

(6) $$\begin{array}{c} I_{0,n}=\frac{I_{sc}}{\exp\left(V_{oc}/aV_{t,n}\right)-1}, \end{array}$$

with *V*_*t*,*n*_ being the thermal voltage at *T*_*n*_. The saturation current *I*_0_ is further improved by including voltage and current coefficients:

(7) $$\begin{array}{c} I_{0}=\frac{I_{sc}+K_{I}\Delta_{T}}{\exp\left(\left(V_{oc}+K_{V}\Delta_{T}\right)/aV_{t}\right)-1}, \end{array}$$

where *K*_*V*_ and *K*_*I*_ are the voltage and the current coefficients, respectively. *V* and *I* are the terminal voltage and current. The relation between *R*_*s*_ and *R*_*p*_ is given by

(8) $$\begin{array}{c} R_{p}=\frac{V_{mp}\left(V_{mp}+I_{mp}R_{s}\right)}{V_{mp}I_{PV}-V_{mp}I_{0}\exp\left[\left(V_{mp}+I_{mp}R_{s}\right)/N_{s}a\right]\left(q/kT\right)+V_{mp}I_{0}-V_{mp}I_{mp}}. \end{array}$$

By using the above equations, the PV model is implemented with MATLAB using the Sim Power system block set.

## 4. Intelligent Maximum Power Tracker

A DC-DC boost converter is used in the first stage of circuit to boost the low voltage from PV array.

The first step in the design of this converter is to determine the duty ratio

(9) $$\begin{array}{c} D=1-\frac{V_{in\left(min\right)}\times ɳ}{V_{out}}. \end{array}$$

The next step is to determine the ripple in the inductor current

(10) $$\begin{array}{c} \Delta I_{L}=\frac{V_{in\left(min\right)}\times D}{f_{s}\times L}. \end{array}$$

The maximum output current is

(11) $$\begin{array}{c} I_{maxout}=\left(\;I_{min}-\frac{\Delta I_{L}}{2}\;\right)\times \left(\;1-D\;\right). \end{array}$$

The maximum switch current is

(12) $$\begin{array}{c} I_{SW\left(max\right)}=\frac{\Delta I_{L}}{2}+\frac{I_{out\left(max\right)}}{1-D}. \end{array}$$

The value of inductor is chosen based on the following equation:

(13) $$\begin{array}{c} L=\frac{V_{in}\times \left(V_{out}-V_{in}\right)}{\Delta I_{L}\times f_{s}\times V_{out}}. \end{array}$$

The output capacitor value for a desired output voltage ripple is

(14) $$\begin{array}{c} C_{out\left(min\right)}=\frac{I_{out\left(max\right)}\times D}{f_{s}\times \Delta V_{out}}. \end{array}$$

To track the maximum power to be transferred from the PV array to the load, maximum power point tracking algorithm is embedded in the first conversion stage. The tracking algorithms are based on the principle that maximum power will be transferred to load when source impedance is equal to load impedance. Out of the many available algorithms, the artificial intelligence technique based FLC for maximum power tracking is considered in this work. Fuzzified natural behavior of change in the insolation level and partial shading effects due to sudden change in climate are considered in the proposed work. Hence, this method proves more advantageous than other methods. This technique can be applied to a system, even if the mathematical model of the system process is not defined for the input-process-output model. For the system under consideration, the fuzzy subsystem fits in deciding the duty ratio of the TSSSBC. To define the control surfaces, each input variable and each output variable are decomposed into a set of fuzzy regions. The control variables considered in the fuzzy set that semantically shows the concept associated with it are error (*e*), change in error (Δ*e*), and the solution variable being the control signal (Δ*d*). Seven membership functions, PB, PM, PS, ZE, NS, NM, and NB, are used to describe them. The formula used to calculate the error (*e*) at *k*th instant is given by

(15) $$\begin{array}{c} e\left(\;k\;\right)=\frac{P\left(k\right)-P\left(k-1\right)}{V\left(k\right)-V\left(k-1\right)}. \end{array}$$

Change in error (Δ*e*) is given by

(16) $$\begin{array}{c} \Delta e=e\left(\;k\;\right)-e\left(\;k-1\;\right). \end{array}$$

Mamdani method was chosen for fuzzy inference. For fuzzification, triangular membership function is chosen for error (*e*), change in error (Δ*e*), and control signal. For the case of the Positive Medium (PM), the membership function is

(17) $$\begin{array}{c} \mu^{PM}\left(\;u\;\right)=\left\{\begin{array}{cc} \max\left\{0.1+\frac{u-0.5}{0.5}\right\}, & \text{if }u\leq 0.5,\\ \max\left\{0.1+\frac{0.5-u}{0.5}\right\}, & \text{otherwise.} \end{array}\right. \end{array}$$

Similar equations are also defined for the rest of the input and output membership functions.

To define the behavior of control surfaces, rule base is framed.

Below is an example for the rule base framed.

*Rule I*. IF error (*e*) is PB AND change in error (Δ*e*) is PB THEN control signal is NB.

Thus, each entry in the table framed is a rule and there are 49 rules that form the knowledge repository of the fuzzy logic controller.

**Figure 3:**
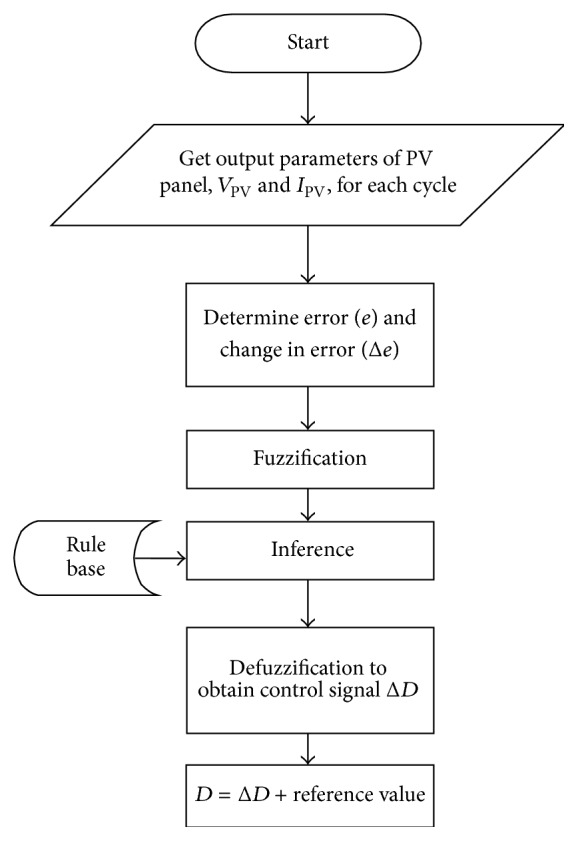
Flowchart of fuzzy logic based power tracker.

The flowchart for the proposed FLC controller is shown in Figure 3. Inputs were fuzzified in the fuzzification process and rule base is framed for this work to produce the required control signal. The centroid method of defuzzification which is the most prevalent and physically appealing of all the defuzzification methods is used to obtain the control signal to drive the pulse for the converter. The formula used is

(18) $$\begin{array}{c} z^{*}=\frac{\int \mu \left(z\right)\cdot zdz}{\int \mu \left(z\right)dz}, \end{array}$$

where *z* ^*∗*^ is the defuzzified value of duty ratio.

Defuzzified signal is the control signal used to vary the duty ratio to extract maximum power from array.

## 5. Design of Three-Stage Soft-Switching Boost Converter

**Figure 4:**
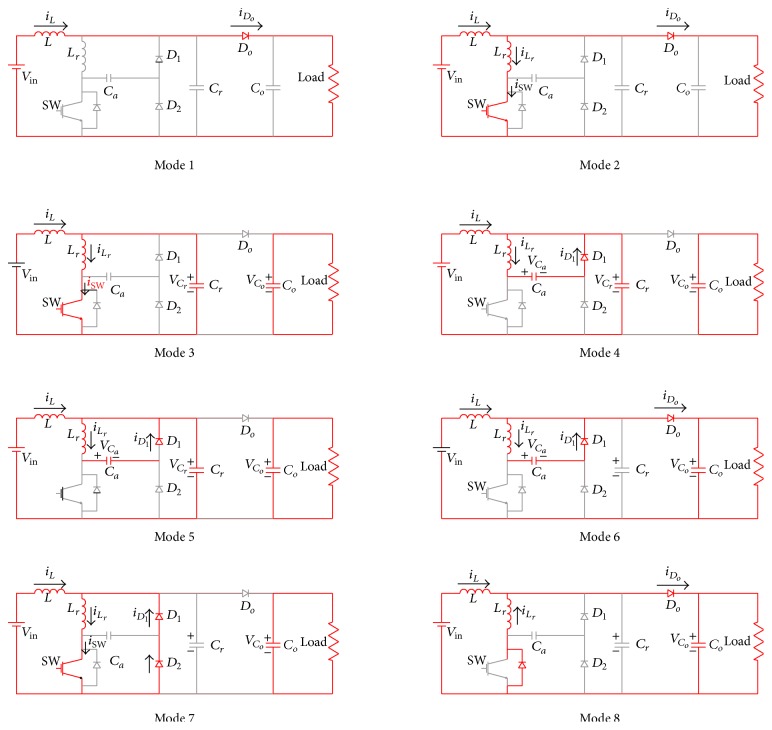
Equivalent circuit diagram of SSSSBC for various modes of operation.

Based on the working of single switch soft switching boost converter (SSSSBC), the three-stage soft switching boost converter (TSSSBC) has been developed and analyzed with eight modes [20].  Figure 4 shows the equivalent circuit diagram for various modes of operation of SSSSBC. For TSSSBC, the same is applicable with a phase shift of 120° for the MOSFET gate pulse in each arm. The formulae for *i*_*L*_(*t*), *i*_*L*_*r*__(*t*), *V*_*C*_*r*__(*t*), and *V*_*C*_*a*__(*t*) during various modes of operation are summarized in Table 2.

**Figure 5:**
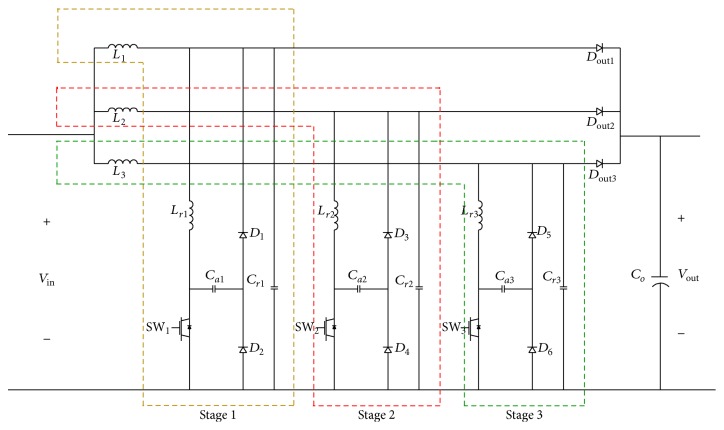
Circuit diagram for three-stage soft-switching boost converter.

The circuit diagram for three-stage soft-switching boost converter (TSSSBC) is shown in Figure 5. In the proposed TSSSBC, the three main inductors (*L*_1_, *L*_2_, and *L*_3_) and resonant inductors (*L*_*r*1_, *L*_*r*2_, and *L*_*r*3_) of reduced magnitudes (1/3rd of the original values) are considered. Driving pulses for the three switches were phase shifted by 120°.

By introducing soft-switching technique, the switching loss is minimized. Hence, the total loss is minimized with the TSSSBC.

## 6. Phase-Locked Loop

**Figure 6:**
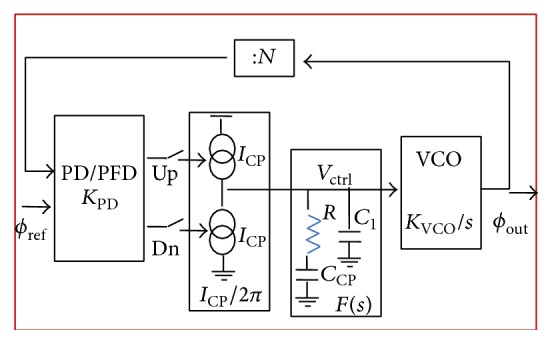
Block diagram of phase-locked loop.

Block diagram of the phase-locked loop (PLL) considered in this work to enhance the performance of the system under consideration is shown in Figure 6.

A PLL is a closed-loop feedback structure that sets fixed phase relationship between its output phase and the phase of a reference. It tracks the phase changes that are within the bandwidth of the PLL.

A PLL comprises several components, namely, phase or phase-frequency detector, charge-pump current, loop filter, voltage-controlled oscillator, and frequency divider.

The open-loop transfer function can be written as

(19) $$\begin{array}{c} H_{open}\left(\;s\;\right)=K_{PFD}\cdot \frac{I_{CP}}{2\pi }\cdot F\left(\;s\;\right)\frac{K_{VCO}}{s}, \end{array}$$

where *K*_PFD_ is the phase-frequency detector gain, *F*(*s*) is the loop filter transfer function, and *K*_VCO_ is the transfer gain of the VCO. The closed-loop transfer function from the input phase to the output phase is a low-pass filter. This low-pass behavior of a PLL is desirable because it rejects input noise frequencies higher than the PLL bandwidth. Similarly, the closed-loop transfer function from the VCO control voltage, *V*_ctrl_, to the output phase is a bandpass filter. It rejects internal noise coupled into *V*_ctrl_ within the PLL bandwidth. Filtering out noise sources by the closed-loop behavior of the PLL forms the baseline for jitter analysis. Noise of the PLL's output clock can be optimally filtered by adjusting the loop bandwidth and peaking in frequency response based on the dominant noise source. The loop bandwidth and peaking are adjustable by varying loop parameters. Natural frequency is proportional to square-root of the loop gain. Damping factor is inversely proportional to zero frequency. By adjusting the zero frequency through the loop filter resistor, *R*, and charge-pump current, *ζ* and *ωn* can be adjusted. To further improve the noise performance, the loop parameters of a PLL should be tuned for minimum output jitter in real system noise conditions.

## 7. Results and Discussions

**Figure 7:**
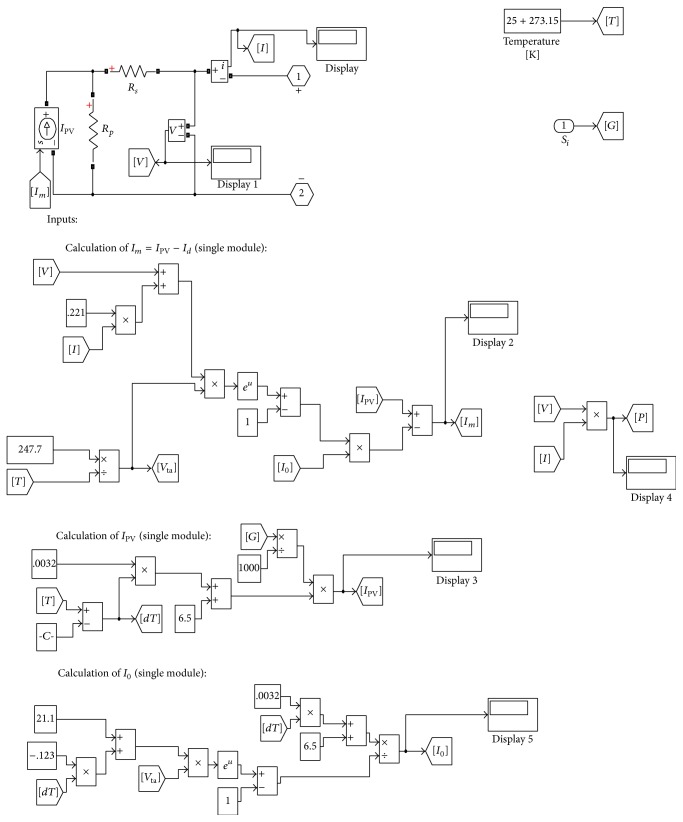
Subsystem for PV array.

**Figure 8:**
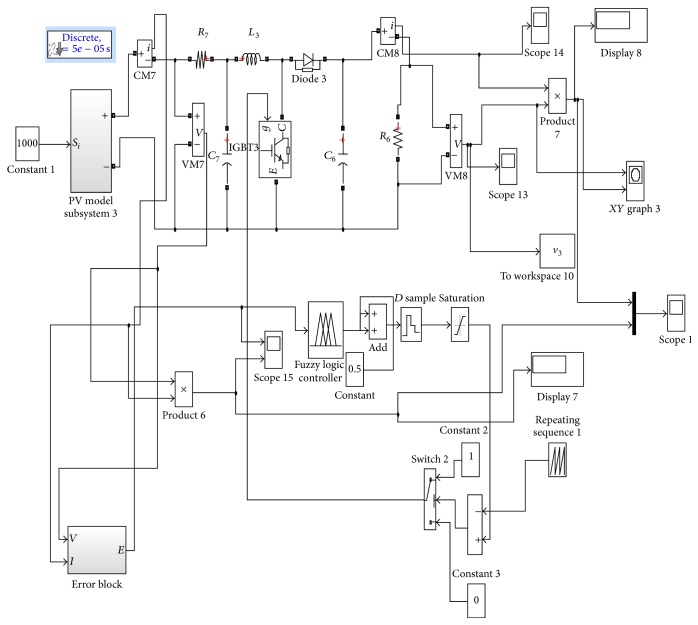
Simulink model for fuzzy logic controller based boost converter.

**Figure 9:**
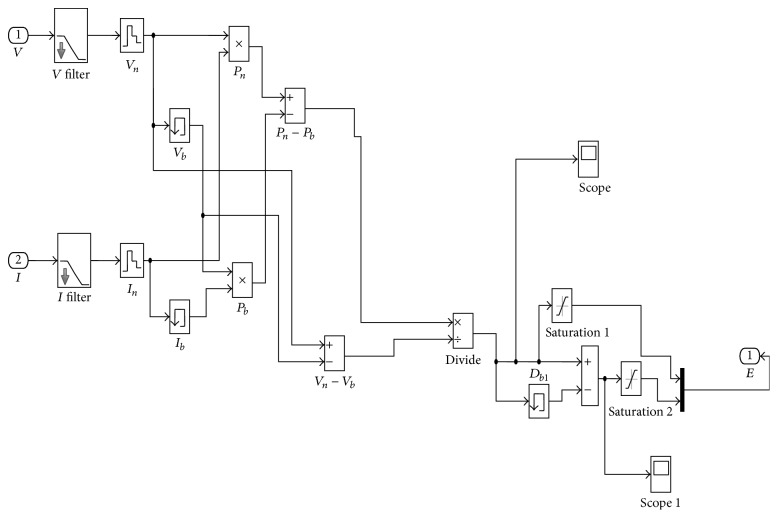
Subsystem of error block.

The developed cascaded converter is simulated using MATLAB/Simulink software package. The subsystem for mathematical model for PV array derived from the equations used in the Simulink model is shown in Figure 7. Simulink model for fuzzy logic controller based boost converter is shown in Figure 8.  Figure 9 shows the error block associated with the system.

The fuzzy logic considered in this work is feedforward network (not feedback from load).

Voltage and current signal sensed from PV array are given as input to filter block and it is sampled. By using multiplier for voltage and current, power is calculated and thereby error and change in error can be calculated using the formulae

(20) ek=Pk−Pk−1Vk−Vk−1,Δe=ek−ek−1.

These two signals are multiplexed and given as input to fuzzy logic block. Fuzzy rule base controls the duty ratio against the variations in error and change in error.

**Figure 10:**
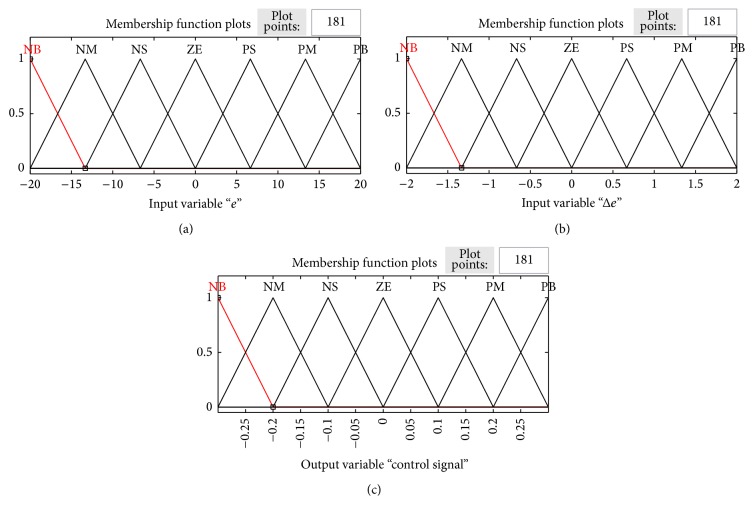
(a) Membership functions of error. (b) Change in error. (c) Control signal.

The control variables considered in the fuzzy set that semantically shows the concept associated with it are error (*e*), change in error (Δ*e*), and the solution variable being the control signal. The membership functions for error (*e*), change in error (Δ*e*), and control signals are shown in Figure 10.

In the proposed design, the universe of discourse for first input, error (*e*) is assigned in terms of seven linguistic variables which are denoted by NB, NM, NS, ZE, PS, PM, and PB. The membership functions for the variable are shown in Figure 10(a).  Figure 10(b) shows the universe of discourse for the second input variable change in error (Δ*e*) which is classified into seven fuzzy sets similar to error.  Figure 10(c) shows the control surface of the output variable control signal.

**Figure 11:**
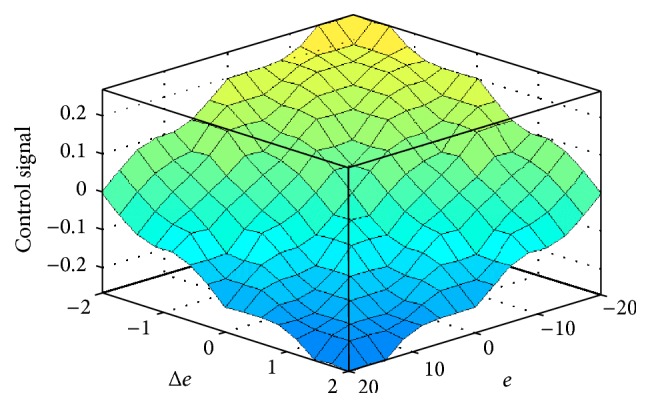
Surface viewer for FLC.

Triangular membership is chosen for both inputs and outputs. The range of values chosen for *e*, Δ*e*, and control signal is (−20, 20), (−2, 2), and (−0.3, 0.3), respectively, based on trial and error. The control signal is added with the reference signal with limits bounded and compared with a ramp signal of desired frequency of operation. The surface viewer is shown in Figure 11. Rule base considered in this work is shown in Table 3.

**Figure 12:**
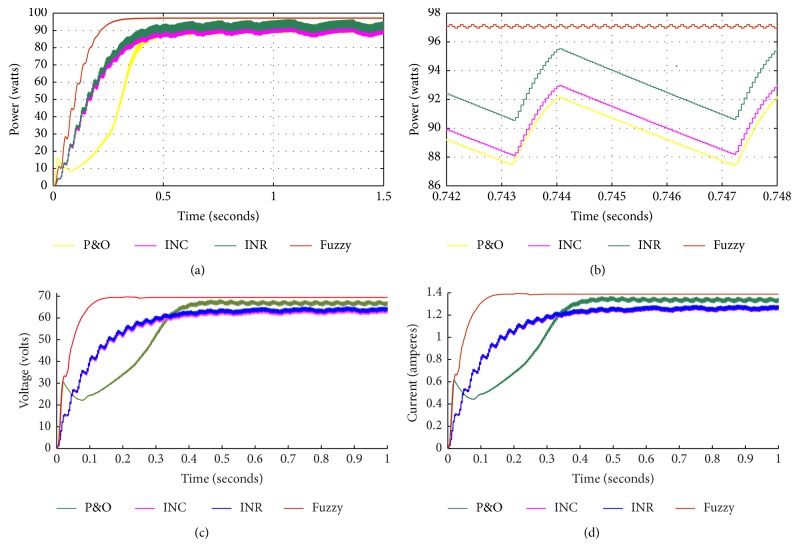
(a) Simulation result of fuzzy based system in comparison with perturb and observe (P&O), incremental conductance (INC), and incremental resistance (INR) method. (b) Zoomed view. (c) Voltage waveform. (d) Current waveform.

**Figure 13:**
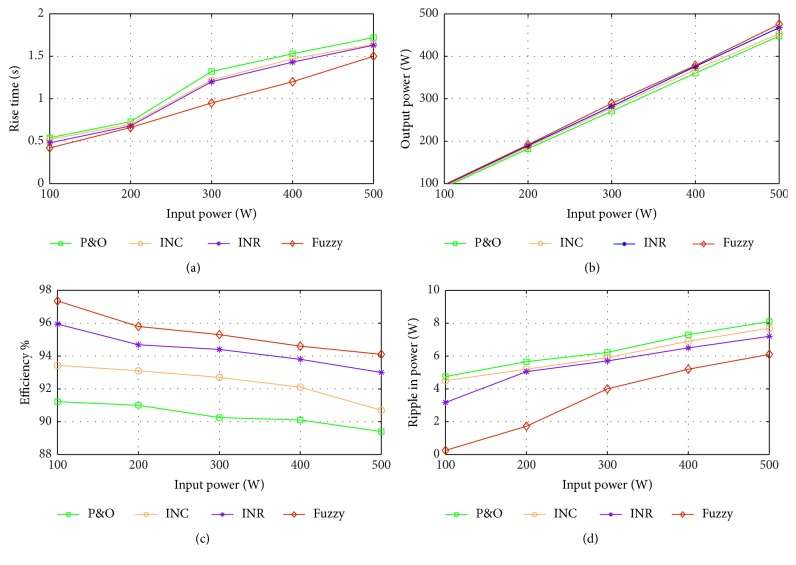
Comparison of performance parameters of different algorithms with respect to (a) rise time, (b) output power, (c) efficiency, and (d) output ripple.

The simulation results of fuzzy based system in comparison with the other algorithms, namely, perturb and observe (P&O), incremental conductance (INC), and incremental resistance (INR) method, are shown in Figure 12. From the graph, it is inferred that maximum power is tracked using the FLC based controller. The performance parameters are tabulated for the simulated results in Table 4 and graphically represented in Figure 13. From the figure, it is observed that overall efficiency of the system obtained is 97.35%. It is, therefore, proved that FLC based power tracking method offers better transient response due to reduced rise time and increased efficiency with reduced ripple as compared with the other algorithms.

**Figure 14:**
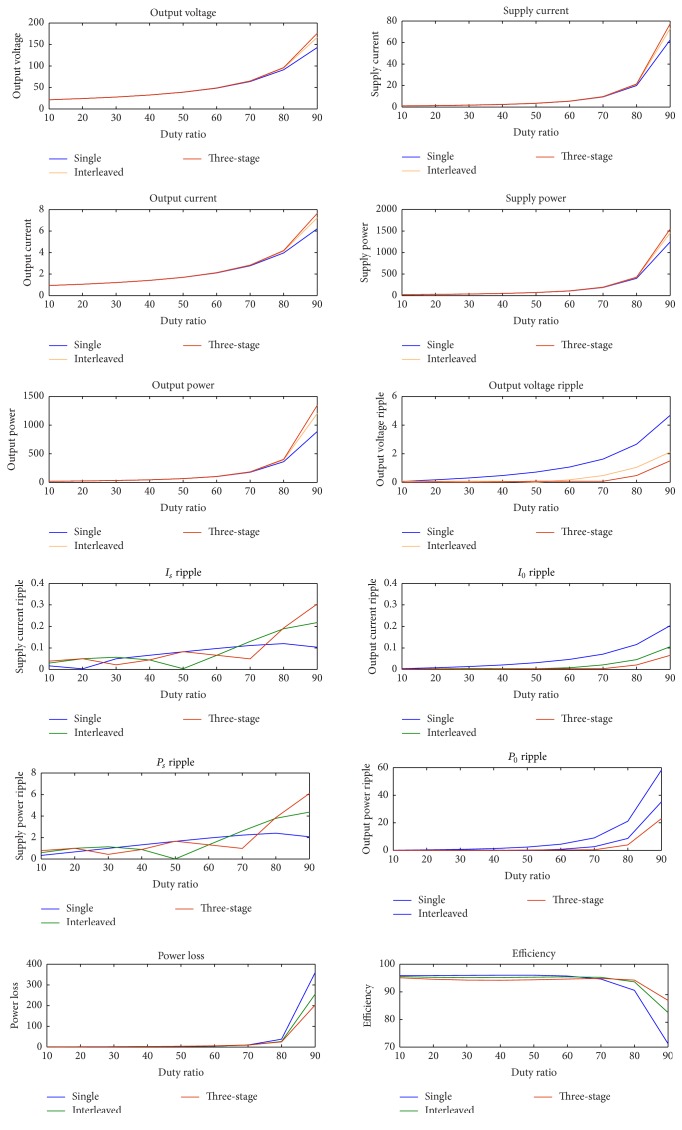
Comparison of performance parameters of single, interleaved, and three-stage converters for varying duty ratio.

In the second stage of the cascaded converter, three-stage soft-switching boost converter is used. The values of the inductor and the capacitor for auxiliary resonant circuit of TSSSBC are chosen so as to design the resonant frequency to be less than the switching frequency. The main inductor rating is 33% of the actual inductor rating used in a single-stage boost converter. The boost converter, interleaved boost converter, and the three-stage boost converter are simulated for the same input voltage and switching frequency and the performances of the three converters were compared and the results are tabulated in Table 5. The comparison with graphical representation is shown in Figure 14. From the tabulated results, it is clear that the ripple content with the three-stage converter is reduced to a greater extent with respect to the single-stage and the interleaved converter for duty ratios from 10% to 90%. In this way, the three-stage boost converter becomes superior to the other ones. A small reduction in efficiency for low duty cycle is due to increased devices and hence increased switching losses for the three-stage converter. Hence, efforts are made to introduce soft-switching technique to the three-stage boost converter to minimize the losses and to improve the performance of the system.

**Figure 15:**
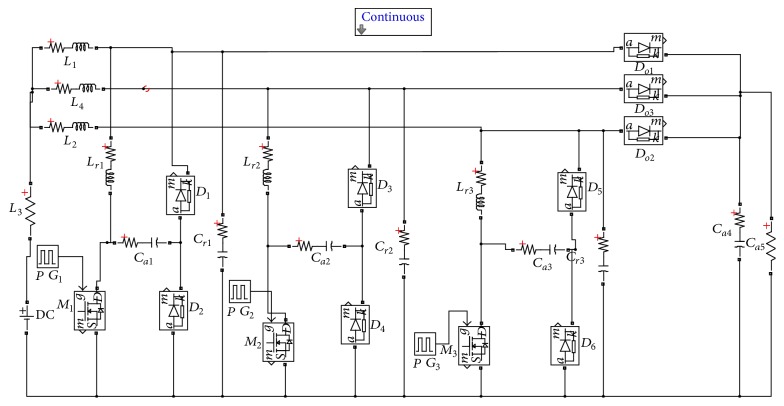
Simulation circuit of three-stage soft-switching boost converter.

**Figure 16:**
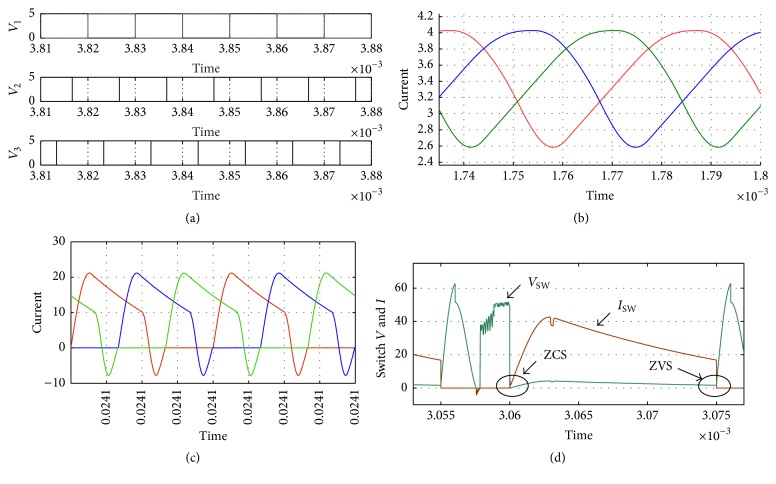
Voltage and current waveforms of TSSSBC. (a) Driving pulses. (b) Main inductors current. (c) Resonant inductors current. (d) Voltage and current through the switch.

The simulated circuit of TSSSBC using Simulink software is shown in Figure 15 and the simulated results are shown in Figure 16. The driving pulses are obtained with 120° phase shift, as shown in Figure 16(a). Figures 16(b) and 16(c) show the main inductor current and the resonant inductor current, respectively. The switching losses are minimized by means of soft-switching technique. In Figure 16(d), the circle pointed out denotes the zero current and zero voltage switching achieved by the converter. Hence, reduction in power loss could be achieved with the proposed topology. The increase in efficiency due to TSSSBC in comparison with simple three-stage boost converter is shown in Table 6.

The specification of active and passive elements used in this work based on the design equation is tabulated in Table 7.

**Figure 17:**
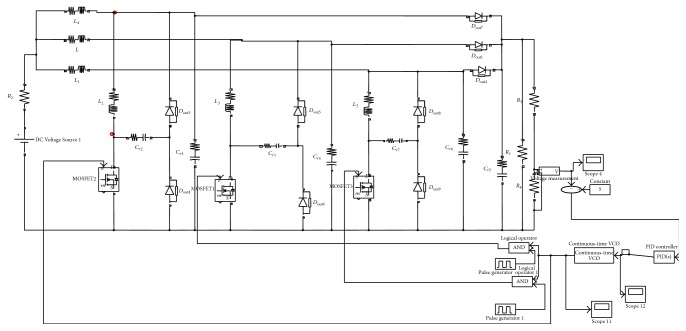
Simulink diagram of TSSSBC with PLL.

**Figure 18:**
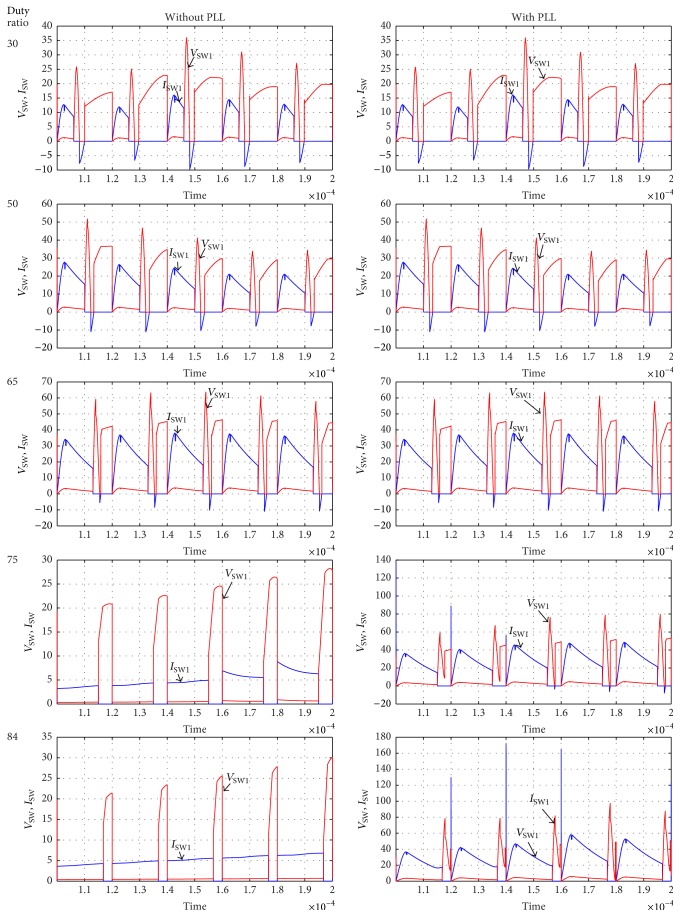
Simulink waveform for different duty ratio.

The simulation circuit of TSSSBC with embedded phase-locked loop for smart grid application is shown in Figure 17. The PLL consists of phase detector, VCO, and loop filter. The waveforms corresponding to switch parameters for various duty ratios are obtained through simulation with the proposed strategy and the conventional circuit without PLL is shown in Figure 18. It clearly depicts that the soft-switching condition exists for the duty ratio range of 0.30 to 0.84 with the proposed system. In the conventional system without PLL, the soft-switching condition occurs only within the duty ratio of 0.30 to 0.72. By including the PLL, the power quality of the system is also improved due to reduced ripple contents.

**Figure 19:**
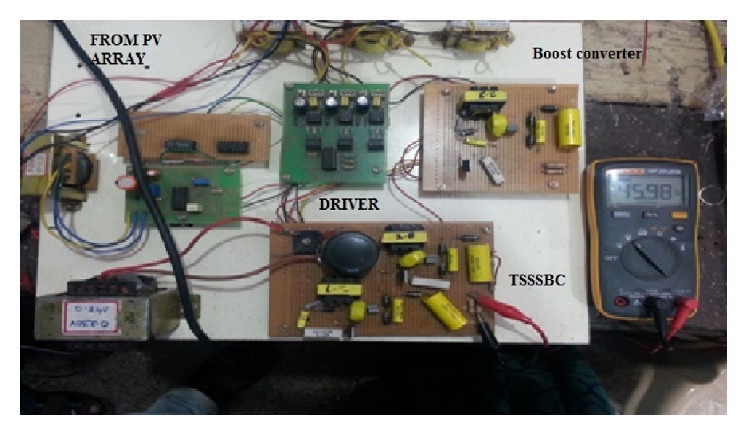
Hardware setup for TSSSBC.

**Figure 20:**
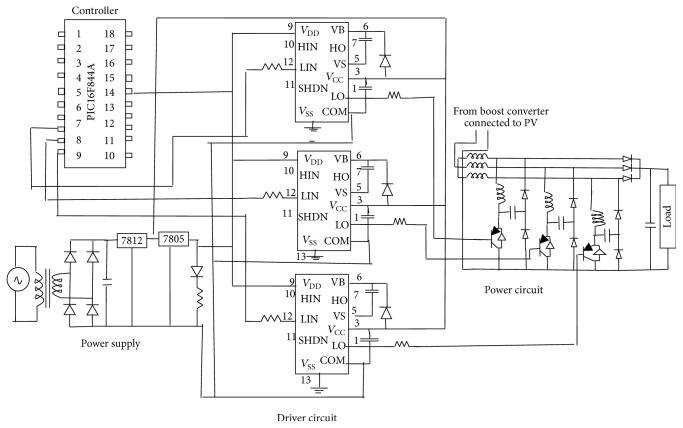
Hardware layout diagram.

The hardware setup is shown in Figure 19 and the layout diagram for the three-stage soft-switching converter is shown in Figure 20.

**Figure 21:**
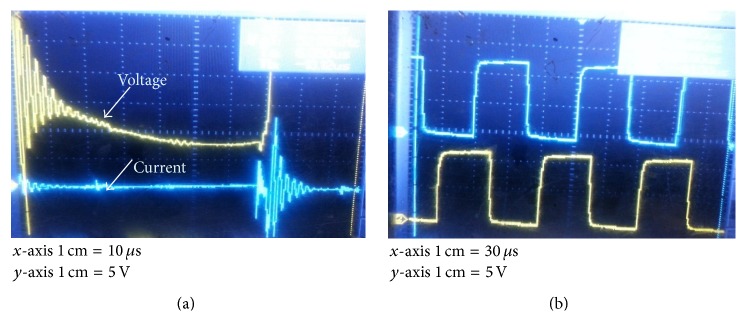
Experimental results of TSSSBC. (a) Voltage across the switch. (b) Pulses for TSSSBC (120° apart).

The PV array is kept at the top floor of the main block. PIC microcontroller 16F84A is used to generate the driving pulses. IR2110 driver IC is used to provide the input at given rating for power MOSFET IRF840. Three resonant inductors and three main inductors along with capacitors form the resonant part in the converter unit. The size of the components is reduced as shown in Figure 19 due to three stages.  Figure 21 shows the waveform obtained from cascaded converter. Input voltage of 18.9 V is applied to the converter by the PV array during noon time. Output voltage of 45.98 V is obtained across the load.

Voltage and current through the switch are shown in Figure 21(a).  Figure 21(b) shows the 120° phase shift between the switching patterns of TSSSBC.

## 8. Conclusions

The cascaded converters with intelligent control techniques are developed to track the maximum power from the photovoltaic (PV) system. Mathematical model of PV array is developed and the same is used for simulation. Sizes of the passive components in the converter are reduced by 33% and the power loss is minimized by adapting the soft-switching technique. The developed model is efficiently used for tracking the maximum power using FLC. The ripple content is minimized and the soft-switching region is increased by embedding PLL. With this arrangement, better transient response is achieved and efficiency of 97.35% is obtained. The experimental verification is done using PIC 16F84A and its results are analyzed. Experimental results are found to be in line with the simulation results. This work can be extended by considering the Genetic Algorithm or particle swarm optimization technique so that the Global Maxima can be obtained for the partial shading condition.

## Figures and Tables

**Table 1:** Monocrystalline PV module specifications at standard test conditions.

Terms (unit)	Value
Open circuit Voltage (V)	21.4
Short circuit current (A)	6.4
Voltage at maximum power (V)	17.4
Current at maximum power (A)	5.75
Maximum power (Wp)	100
Tolerance (%)	+5
Maximum system voltage (V)	1000

**Table 2:** Current and voltage equations of SSSSBC for various modes.

Modes	*i*_*L*_(*t*)	*i*_*L*_*r*__(*t*)	*V*_*C*_*r*__(*t*)	*V*_*C*_*a*__(*t*)
Mode 1	$i_{L}(t_{0})-\frac{\left(V_{0}-V_{in}\right)}{L}t$	0	*V*_0_	0

Mode 2	$i_{1}-\frac{\left(V_{0}-V_{in}\right)}{L}t$	$\frac{V_{0}}{L_{r}}t$	*V*_0_	0

Mode 3	*I*_min_	0	$I_{min}+\frac{V_{0}}{Z_{r}}\sin\omega_{r}t$	*V*_0_cos⁡ω_*r*_ *t*

Mode 4	$I_{min}+\frac{V_{in}}{L}t$	*I*_2_	0	0

Mode 5	*I*_max_	*I*_2_cos⁡ω_*a*_ *t*	$\frac{I_{2}}{C_{a}}$	*Z*_*r*_ *I*_2_sin⁡ω_*a*_ *t*

Mode 6	$I_{3}-\left(\frac{V_{0}-V_{in}}{L}\right)*t$	*I*_2_cos⁡ω_*a*_ *t*	*V*_0_	*Z*_*r*_ *I*_2_sin⁡ω_*a*_ *t*

Mode 7	$I_{3}-\left(\frac{V_{0}-V_{in}}{L}\right)*t$	$\left(\frac{V_{0}}{Z_{a}}-I_{2}\right)\sin\omega_{a}t$	*V*_0_	*V*_2_

Mode 8	$I_{4}+\left(\frac{V_{0}-V_{in}}{L}\right)*t$	$I_{s}-\left(\frac{V_{0}}{L_{r}}\right)*t$	*V*_0_	0

**Table 3:** Rule base for FLC.

*e*	Δ*e*						
	NB	NM	NS	ZE	PS	PM	PB
NB	PB	PB	PB	PM	PM	PM	PS
NM	PB	PB	PM	PM	PM	PS	ZE
NS	PB	PB	PM	PM	PS	PS	ZE
ZE	PB	PM	PS	ZE	ZE	ZE	NS
PS	PM	PM	PS	PS	ZE	S	NS
PM	PM	PM	PS	ZE	NS	NS	NM
PB	PS	PS	ZE	NS	NM	NM	NB

**Table 4:** Performance parameters of different algorithms.

Input power (W)	Rise time (s)				Settling time				Output power				Ripple				Efficiency			
	P&O	INC	INR	Fuzzy	P&O	INC	INR	Fuzzy	P&O	INC	INR	Fuzzy	P&O	INC	INR	Fuzzy	P&O	INC	INR	Fuzzy
100	0.54	0.52	0.48	0.42	0.56	0.54	0.49	0.43	91.22	93.43	95.95	97.35	4.75	4.5	3.17	0.25	91.22	93.4	96	97.35
200	0.73	0.7	0.68	0.66	0.76	0.72	0.71	0.67	182	186.2	189.4	191.6	5.66	5.2	5.05	1.72	91	93.1	94.7	95.8
300	1.32	1.22	1.2	0.95	1.35	1.25	1.23	0.96	270.75	278.1	282	289.2	6.22	5.9	5.7	4	90.25	92.7	94.4	95.3
400	1.53	1.47	1.43	1.2	1.55	1.48	1.44	1.22	360.4	368.4	376	378.4	7.3	6.9	6.5	5.2	90.1	92.1	93.8	94.6
500	1.72	1.64	1.63	1.5	1.76	1.67	1.64	1.54	447.7	453.5	467.5	475.5	8.1	7.7	7.2	6.1	89.4	90.7	93	94.1

**Table 5:** Comparison of single, interleaved, and three-stage converters.

	*V*_0_	*I*_*s*_	*I*_0_	*P*_*s*_	*P*_0_	Δ*V*_0_	Δ*I*_*s*_	Δ*I*_0_	Δ*P*_*s*_	Δ*P*_0_	Power loss	Efficiency
Single-stage												
10%	21.41	1.04	0.93	20.78	19.93	0.08	0.02	0.00	0.33	0.15	0.85	95.91
20%	24.16	1.32	1.05	26.47	25.38	0.18	0.00	0.01	0.66	0.38	1.08	95.91
30%	27.69	1.74	1.20	34.74	33.34	0.31	0.05	0.01	0.99	0.75	1.40	95.96
40%	32.37	2.37	1.41	47.44	45.55	0.48	0.07	0.02	1.32	1.36	1.89	96.02
50%	38.85	3.42	1.69	68.35	65.62	0.72	0.08	0.03	1.64	2.43	2.73	96.01
60%	48.39	5.32	2.10	106.36	101.81	1.07	0.10	0.05	1.95	4.51	4.55	95.72
70%	63.67	93.16	2.77	186.33	176.29	1.63	0.11	0.07	2.22	9.06	10.04	94.61
80%	91.18	19.96	3.96	399.28	361.51	2.67	0.12	0.12	2.40	21.14	37.76	90.54
90%	142.95	62.39	6.22	1247.89	888.70	4.68	0.10	0.20	2.06	58.20	359.19	71.22

Two-stage												
10%	21.38	1.04	0.93	20.88	19.94	0.10	0.03	0.001	0.59	0.07	0.94	95.51
20%	24.18	1.33	1.05	26.69	25.42	0.07	0.05	0.001	1.00	0.15	1.27	95.24
30%	27.73	1.76	1.21	35.14	33.43	0.09	0.06	0.001	1.14	0.22	1.71	95.14
40%	32.45	2.41	1.41	48.10	45.78	0.08	0.04	0.001	0.88	0.23	2.32	95.17
50%	39.02	3.47	1.70	69.46	66.19	0.01	0.00	0.001	0.00	0.02	3.26	95.30
60%	48.78	5.42	2.12	108.43	103.45	0.18	0.07	0.01	1.32	0.78	4.97	95.41
70%	64.73	9.56	2.81	191.29	182.19	0.48	0.13	0.02	2.60	2.71	9.10	95.24
80%	94.98	20.95	4.13	419.04	392.24	1.05	0.19	0.05	3.79	8.68	26.80	93.60
90%	166.48	72.96	7.23	1458.08	1202.82	2.11	0.22	0.11	4.36	35.18	255.26	82.49

Three-stage												
10%	21.42	1.05	0.93	20.97	19.94	0.02	0.04	0.001	0.78	0.04	1.03	95.09
20%	24.19	1.35	1.05	26.90	25.43	0.03	0.05	0.001	1.00	0.07	1.47	94.54
30%	27.74	1.78	1.21	35.50	33.46	0.02	0.02	0.001	0.43	0.04	2.04	94.24
40%	32.47	2.43	1.41	48.67	45.85	0.04	0.04	0.001	0.89	0.11	2.83	94.20
50%	39.08	3.52	1.70	70.36	66.39	0.08	0.08	0.001	1.66	0.29	3.97	94.35
60%	48.92	5.50	2.13	109.93	104.03	0.08	0.07	0.001	1.32	0.35	5.90	94.63
70%	65.09	9.71	2.83	194.22	184.26	0.08	0.05	0.001	0.98	0.46	9.97	94.87
80%	96.32	21.40	4.19	428.07	403.36	0.48	0.19	0.02	3.86	4.01	24.71	94.23
90%	175.91	77.43	7.65	1548.52	1345.42	1.51	0.30	0.07	6.10	23.06	203.10	86.88

**Table 6:** Comparison of three-stage converter and TSSSBC.

Sl. number	Duty ratio	Efficiency of three-stage converter	Efficiency of the proposed TSSSBC
1	10%	95.09	96.43
3	30%	94.24	95.03
5	50%	94.35	95.01
7	70%	94.87	95.74
8	80%	94.23	95.02

**Table 7:** Specifications of the components.

Parameters	Symbol	Value	Unit
Main inductor	*L*_1_, *L*_2_, *L*_3_	187.6	*μ*H
Resonant inductor	*L*_*r*1_, *L*_*r*2_	6	*μ*H
Auxiliary capacitor	*C*_*a*1_, *C*_*a*2_, *C*_*a*3_	15.5	nF
Resonant capacitor	*C*_*r*1_, *C*_*r*2_, *C*_*r*3_	405	nF
Output capacitor	*C*_*o*_	10	*μ*F
